# Diffusion MRI tractography with along-tract profiling reveals subtle neurodevelopmental differences between moderate and late preterm infants

**DOI:** 10.1016/j.ejrad.2025.112098

**Published:** 2025-04-05

**Authors:** Anouk S. Verschuur, Chantal M.W. Tax, Ingrid M. Nijholt, Gerda van Wezel-Meijler, Leonora Hendson, Hussein Zein, Jeanne Scotland, Regan King, Khorshid Mohammad, Martijn F. Boomsma, Alexander Leemans, Lara M. Leijser

**Affiliations:** a Department of Radiology, Isala Hospital, Dokter van Heesweg 2, 8025 AB Zwolle, The Netherlands; b Image Sciences Institute, University Medical Center Utrecht, Heidelberglaan 100, 3584 CX Utrecht, The Netherlands; c Department of Pediatrics, Section of Newborn Critical Care, University of Calgary, 3330 Hospital Drive NW, Calgary AB T2N 4N1, Canada; d CUBRIC, School of Physics and Astronomy, Cardiff University, CF10 3AT Cardiff, United Kingdom; e Section of Newborn Critical Care, Alberta Health Services, 4520 16 Avenue NW, Calgary AB T3B 0M6, Canada; f Division of Imaging and Oncology, University Medical Center Utrecht, Heidelberglaan 100, 3584 CX Utrecht, The Netherlands; g Department of Neonatology, Isala Women and Children’s Hospital, Dokter van Heesweg 2, 8025 AB Zwolle, The Netherlands

**Keywords:** Tractography, Moderate-late, preterm, Diffusion MRI, Tractometry

## Abstract

**Purpose:**

Moderately preterm (MP) infants (32–33 weeks’ gestation) are at increased risk for developmental problems compared to late preterm (LP) infants (34–36 weeks’ gestation). Fiber bundle tractography remains an unexplored avenue to understanding this risk-difference between MP and LP infants. This study aimed to examine along-tract profile differences between MP and LP infants at term-equivalent age (TEA).

**Methods:**

Ninety-five infants (31 MP and 64 LP), born between November 2020 and March 2023, underwent MRI around TEA (40–44 weeks postmenstrual age). MRI included T2-weighted imaging and diffusion MRI (dMRI) with b-values 800 and 2000 s/mm^2^ (single shell). dMRI scans were preprocessed to reduce common artifacts. For all infants, 15 fiber bundles were reconstructed using TractSeg and along-tract profiles, expressed as fractional anisotropy (FA) and mean diffusivity (MD), and were compared between MP and LP infants using tractometry. *Results:* Reconstructions with TractSeg demonstrated shape, position, and orientation of fiber bundles consistent with known neuroanatomy. FA and MD profiles were not significantly different between MP and LP infants. However, alternating trends towards along-tract profile differences between MP and LP infants were observed for multiple bundles. Wide 95% confidence intervals indicated substantial variability in fiber bundle organization within groups.

**Conclusion:**

Although not significant, along-tract differences between MP and LP infants suggest subtle alterations in white matter maturation. These findings indicate along-tract variability as potential focus for future research aimed at uncovering the mechanisms underlying early maturational differences and their potential role in later neurodevelopmental challenges encountered in moderate-late preterm infants.

## 1 Introduction

Worldwide, an estimated 13 million infants born prematurely (<37 weeks’ gestation) each year face an increased risk of life-long problems with neurodevelopment and social functioning [1–3]. A largely understudied population, that comprises the vast majority of preterm infants (85 %), are born moderate to late preterm (MLPT; 32–36 weeks’ gestation) [4]. Recent studies have reported more frequent attention problems, hyperactivity, socio-emotional challenges, and impaired motor and cognitive development in children born MLPT as compared to those born full term [5–9], with moderate preterm infants (MP; 32^+0^-33^+6^ weeks’ gestation) being more frequently affected than late preterm (LP; 34^+0^-36^+6^ weeks’ gestation) infants [6]. These developmental problems may not only restrict the quality of life for these infants and families, but, given population size, also have major implications for healthcare systems and society. Early identification of MLPT infants at risk for neurodevelopmental problems would enable targeted early intervention to improve outcome. At present, the early neurobiological changes underlying the neurodevelopmental problems in MLPT infants remain unclear. A better understanding of the anticipated subtle brain microstructural variations, and potential differences between MP and LP infants, may help elucidate the mechanisms of the varying neuro-developmental vulnerabilities across the MLPT population. This may enable early identification of the highest risk MLPT infants and the development of novel, individualized neuroprotective strategies targeted to their specific needs.

A dedicated method to study the complex architecture and microstructural variations of the brain is diffusion MRI (dMRI) [10]. Tractography, the virtual reconstruction of white matter fiber bundles, can be used to study tract specific developmental differences between populations. Tractography from dMRI has been widely applied in the young infant brain to study early brain development [11]. However, most of these studies used diffusion tensor imaging (DTI) for fiber bundle reconstruction [12]. DTI may not fully capture the brain’s architectural complexity as DTI only allows for reconstruction of one white matter fiber direction per voxel [13]. Advanced techniques that more accurately reconstruct crossing fiber bundles may elucidate subtle differences between neonatal brains and provide insights into factors contributing to suboptimal neurodevelopmental outcomes.

We recently demonstrated the feasibility of reconstructing crossing fiber bundles in neonates [14], while also highlighting the variability in reconstruction imposed by differing image processing and reconstruction settings. Deep learning-based toolboxes, such as TractSeg, may help reduce this variability and improve reproducibility of complex fiber bundle reconstructions. Furthermore, along-tract profiling – analyzing diffusion measures along specific tract segments instead of averaging across the entire fiber bundle – provides a more anatomically detailed assessment of inter-individual differences in diffusion. Commonly investigated diffusion measures, fractional anisotropy (FA) and mean diffusivity (MD), reflect the microstructural architecture of the imaged tissues [10]. As the brain matures, increases in FA and decreases in MD are indicative of ongoing structural development. To the best of our knowledge, deep learning-based toolboxes like TractSeg have not been tested yet for tract reconstruction from neonatal dMRI data. In addition, potential fiber bundle tractography differences and along-tract variability between MP and LP infants have yet to be studied.

With this study, using TractSeg to reconstruct white matter fiber bundles from dMRI acquired at term-equivalent age (TEA), we aimed to assess early microstructural brain developmental differences between MP and LP infants. Specifically, we aimed to assess accuracy of tractography output and compare along-tract diffusion profiles between MP and LP infants using tractometry.

## 2 Methods

### 2.1 Population

The Canadian ‘Brain Imaging in Moderate-Late Preterm infants’ (BIMP) cohort of MLPT infants (32^+0^-35^+6^ weeks’ gestation) was prospectively recruited from the neonatal intensive care units at Rockyview General Hospital and Peter Lougheed Centre, Calgary, Canada, between November 2020 and March 2023 (ethics approval: REB19-1194). Congenital malformations of the central nervous system, chromosomal disorders, inborn errors of metabolism, congenital infections, central nervous system infections, or parents unable to provide written informed consent in English were reasons to exclude infants from participating in the study. Signed parental informed consent was obtained for infants to participate in the BIMP−study. None of the included infants had brain lesions as assessed from conventional MRI and cranial ultrasound by experts (LML and GvWM). Infant clinical characteristics were collected: GA (weeks), sex, plurality, admission level, weight (grams; at birth and at time of MRI), head circumference (cm; at birth and at time of MRI) and postmenstrual age (PMA; weeks) at time of MRI, small for gestational age (below third percentile), need for continuous positive airway pressure (CPAP) support, suspicion of early onset sepsis, meningitis, bronchopulmonary dysplasia, necrotizing enterocolitis, hemodynamically significant patent ductus arteriosus, and hypoxicischemic encephalopathy.

### 2.2 Data acquisition

Infants underwent TEA (40–44 weeks PMA) MRI at the Alberta Children’s Hospital using a research-dedicated 3 Tesla General Electric MR750W system, managed by the Child and Adolescent Imaging Research (CAIR) program at the University of Calgary. Natural sleep was induced with the feed and sleep technique, and a vacuum bag further immobilized the infant. Earmuffs and headphones provided hearing protection [15].

T2-weighted and dMRI scans were used for this study. T2-weighted scans were acquired with axial fast spin echo, repetition time =4400 ms, echo time =120 ms, flip angle =111 degrees, acquisition matrix =320x320, reconstruction matrix =512x512, field of view =19.2 cm, pixel spacing =0.375x0.375 mm, slice thickness =3 mm [0.4 mm gap]. dMRI scans were acquired with pulsed-gradient spin echo echo planar imaging (PGSE EPI), b-value =800 and 2000 s/mm^2^ (referred to as b800 and b2000 hereafter) acquired separately (i.e., single shell), 45 non-collinear gradient directions, 5b = 0 s/mm^2^ images per dMRI scan, repetition time = 7000 ms, echo time (b800/b2000) = 81.4 ms/97.9 ms, 49 slices, acquisition matrix = 100x100, reconstruction matrix =256x256, acquired voxel size = 2x2x2mm.

### 2.3 MRI processing

The brain extraction tool (BET) from the FMRIB Software Library (FSL; version 6.0.3, Oxford University, UK) [16] was used for skull stripping the T2-weighted MRI scans, and subsequent manual correction was applied when extracerebral tissue was still present.

MRTrix3 (version 3.0.2) and Advanced Normalization Tools (ANTs; version 2.3.5) were used for preprocessing the dMRI scans [17,18]. The b800 and b2000 scan were concatenated for preprocessing. Preprocessing with MRTrix3 included denoising (with patch size =7) [19], correction for Gibbs ringing (default settings) [20], motion correction (with correction for slice-to-volume misalignment [21] and outlier handling [22]; eddy options included: slm =linear, mporder =6, s2v_niter =5, s2v_lambda =1 s2v_interp =trilinear), eddy induced distortion correction [23], and bias correction (default settings) [24,25]. Subsequently, ANTs registration was used to correct echo planar imaging (EPI) distortions by rigid registration of the T2-weighted images to the mean b0-image, followed by anterior-posterior affine registration (BSplineSyN) of the mean b0-image to the registered T2-weighted images. The b0 transformation parameters were then applied to the full 4-dimensional dMRI volume.

### 2.4 TractSeg and tractometry

**Fig. 1:**
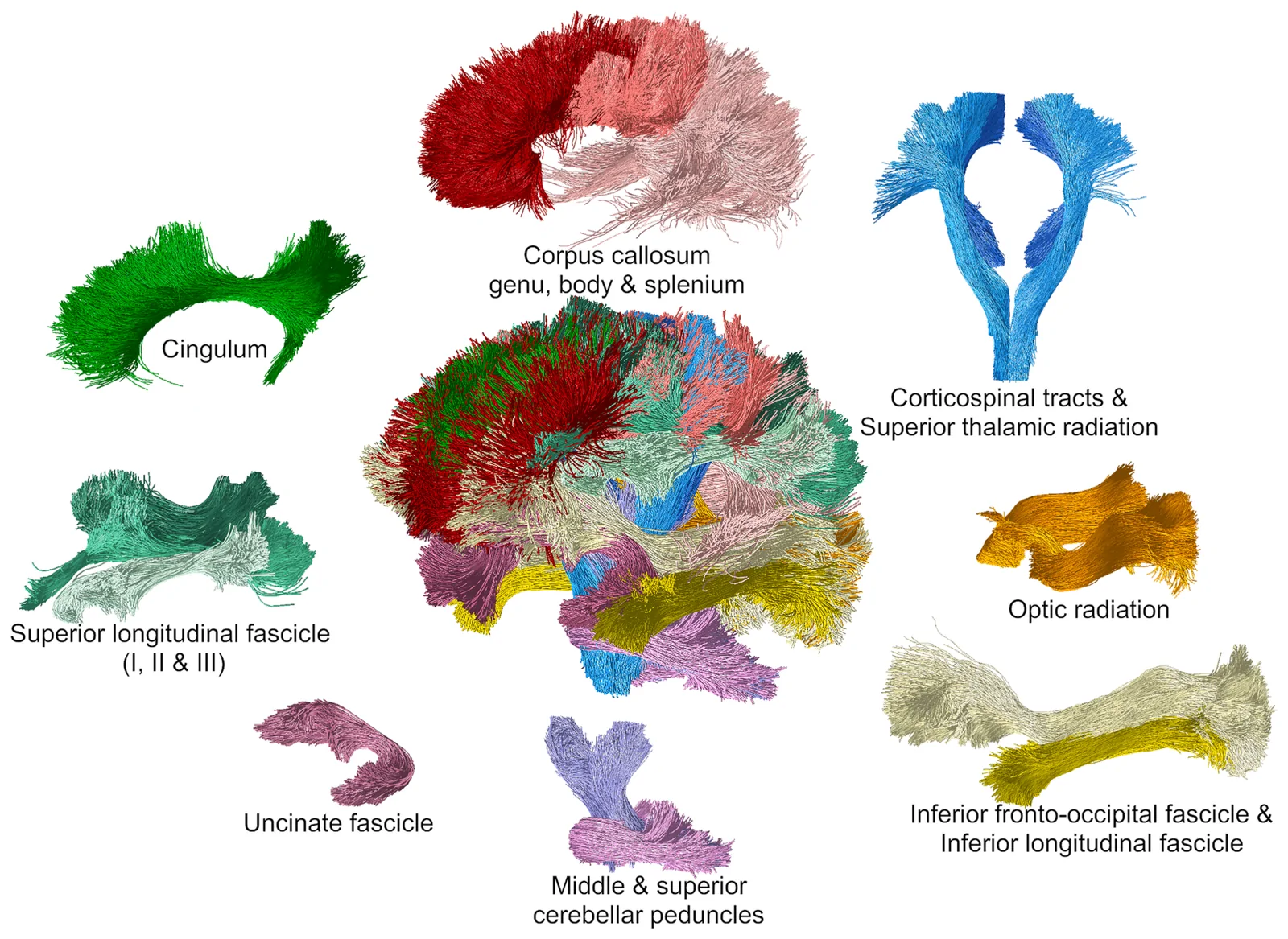
Selected fiber bundles included in the analyses, shown in their anatomical positions within the brain (center) and isolated visualizations as reconstructed with TractSeg.

Tractography was performed on b2000 scans using TractSeg with standard settings [26]. In short, a convolutional neural network, trained on adult dMRI data from the human connectome project, consecutively created tract segmentations, segmentations for bundle beginnings and endings, tract orientation maps, and fiber bundle streamline reconstructions with probabilistic tractography and 5000 streamlines for each tract [26]. For the purpose of this study, we selected 15 fiber bundles from the 72 extracted by TractSeg, combining bilateral bundles and dividing the corpus callosum into three parts. The selected 15 bundles represent major connections that are stimulated from an early age, encompassing key functional domains such as vision, motor function, speech/language processing, and cognition. Selected fiber bundles were: corpus callosum genu (i.e., rostrum and genu), body (i.e., rostral body, anterior midbody and posterior midbody) and splenium (i. e., isthmus and splenium), cingulum bundle, middle cerebellar peduncle, superior cerebellar peduncle, inferior longitudinal fascicle, inferior fronto-occipital fascicle, optic radiation, superior longitudinal fascicle I, II and III, superior thalamic radiation, and uncinate fascicle (Fig. 1). Corticospinal tracts were included as control. Bilateral bundles were combined to reduce the number of comparisons, anticipating no left–right differences in fiber bundle development between hemispheres in this cohort without overt brain injury (such as large intraventricular hemorrhages, periventricular infarction or stroke). Fiber bundle reconstructions were visually quality checked to ensure appropriate reconstruction. The visual quality check comprised of assessment of the overall tract shape, symmetry, and overlap with anatomical structures on the b0 dMRI.

After reconstruction, tractometry [27,28] was used for along-tract profiling of the diffusion measures FA and MD. FA, the most widely used measure, describes the directional coherence of water diffusion in white matter and is associated with brain maturational processes such as myelination, axon packing, relative membrane permeability to water, inter-axonal structure and tissue water content [29]. MD, on the other hand, measures the overall degree of diffusion and is typically lower in areas with high tissue complexity due to diffusion hindrance [29]. The choice of FA and MD was based on their widespread use in neonatal research, allowing for comparability with previous studies. Additionally, other diffusion metrics were not technically feasible or reliable given the available data, limited to single shell acquisitions, common in neonatal imaging. Fiber tracts were divided into 20 equal segments across each tract. The number of segments, initially set to 100 as the default in tractometry, was reduced to 20 to better suit the smaller brain size of neonates. The two end segments of each bundle were excluded to remove potential influences of partial volume effects (e.g., gray matter). FA and MD maps were generated from the b0 and b800 scans to minimize the effects of non-Gaussian diffusion behavior. Otherwise, standard tractometry settings were applied.

### 2.5 Analyses

Infant characteristics were calculated for included MP and LP infants and excluded infants. Means (with standard deviation [SD]) or medians (with range) were calculated for continuous variables, and counts (with percentage) for categorical variables. Characteristics were compared between included MP and LP infants and between included and excluded infants using the chi-squared test (dichotomous variables), independent *t*-test (continuous variables with normal distribution) or Mann-Whitney *U* test (continuous variables with non-normal distribution).

Tractometry utilizes a 2-sample independent *t*-test, comparing diffusion measures between MP and LP infants for each segment of all 15 bundles [27]. Prior to analyses, normality and equal variances between MP and LP infants were confirmed per bundle and segment. Additionally, PMA at time of MRI was tested as a potential confounder, which was ruled out. *T*-test analyses were corrected for multiple comparisons using the permutation based multiple comparison correction (with n = 5000 repetitions) [30]. Statistical significance was set to p < 0.05 for descriptive analyses.

## 3 Results

### 3.1 Infant characteristics

**Fig. 2:**
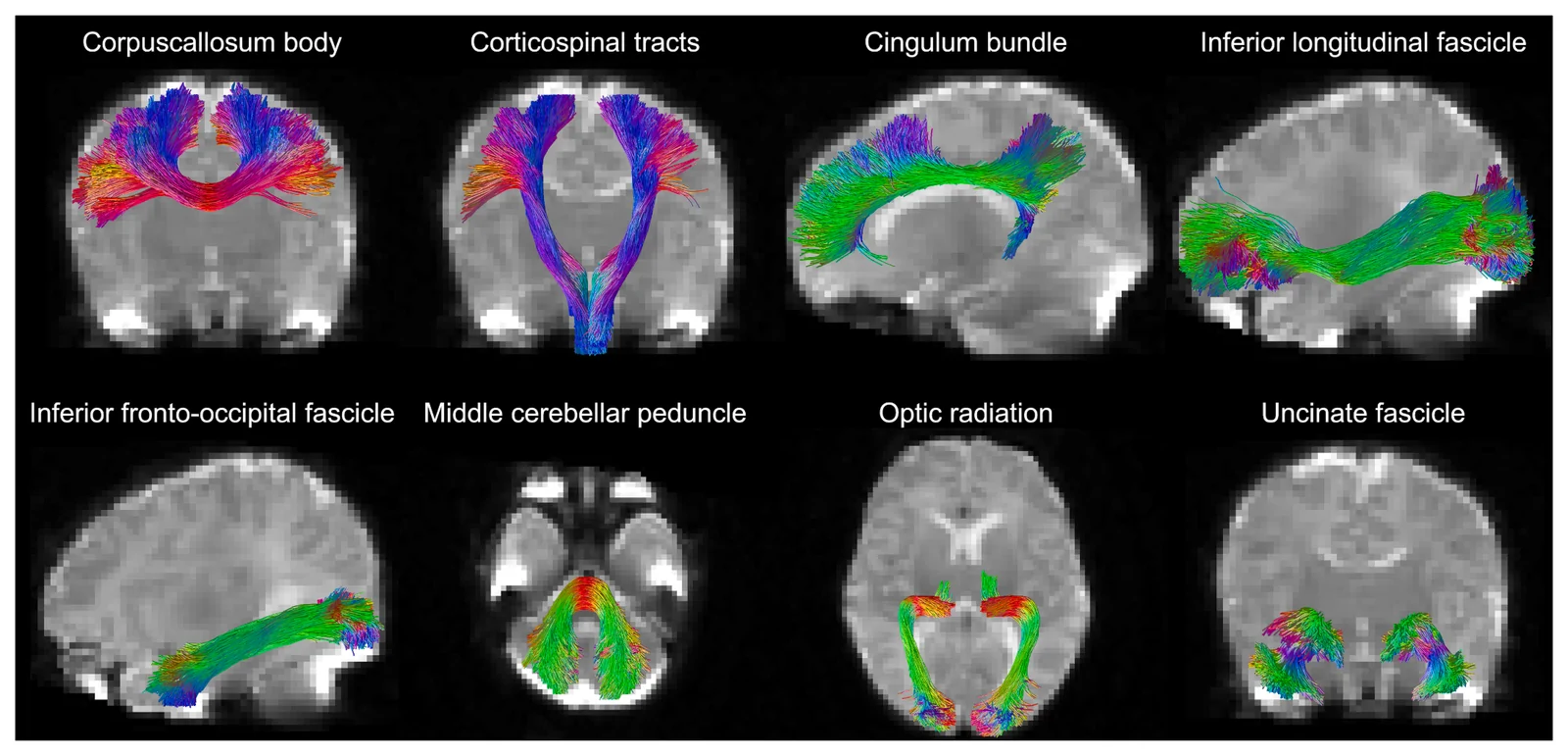
Inclusion flowchart.

A total of 138 MLPT infants were recruited after birth, of whom 121 underwent MRI around TEA. Of these, 105 (76 %) had both b800 and b2000 scans (Fig. 2). Infants with incomplete or no b800 (n = 3) or b2000 (n = 13) scan were excluded from analyses. After visual quality checking of the TractSeg outputs, another ten infants were excluded. In total, the scans of 95 infants (31 MP and 64 LP) were included in the analyses (Fig. 2). Significant differences between MP and LP infants were found for GA, weight and head circumference at birth, and for CPAP need, suspicion of early onset sepsis, and bronchopulmonary dysplasia, no other significant differences in infant characteristics were found between MP and LP infants; no differences in characteristics were found between included and excluded MLPT infants (Table 1).

### 3.2 Tractography and tractometry

**Fig. 3:**
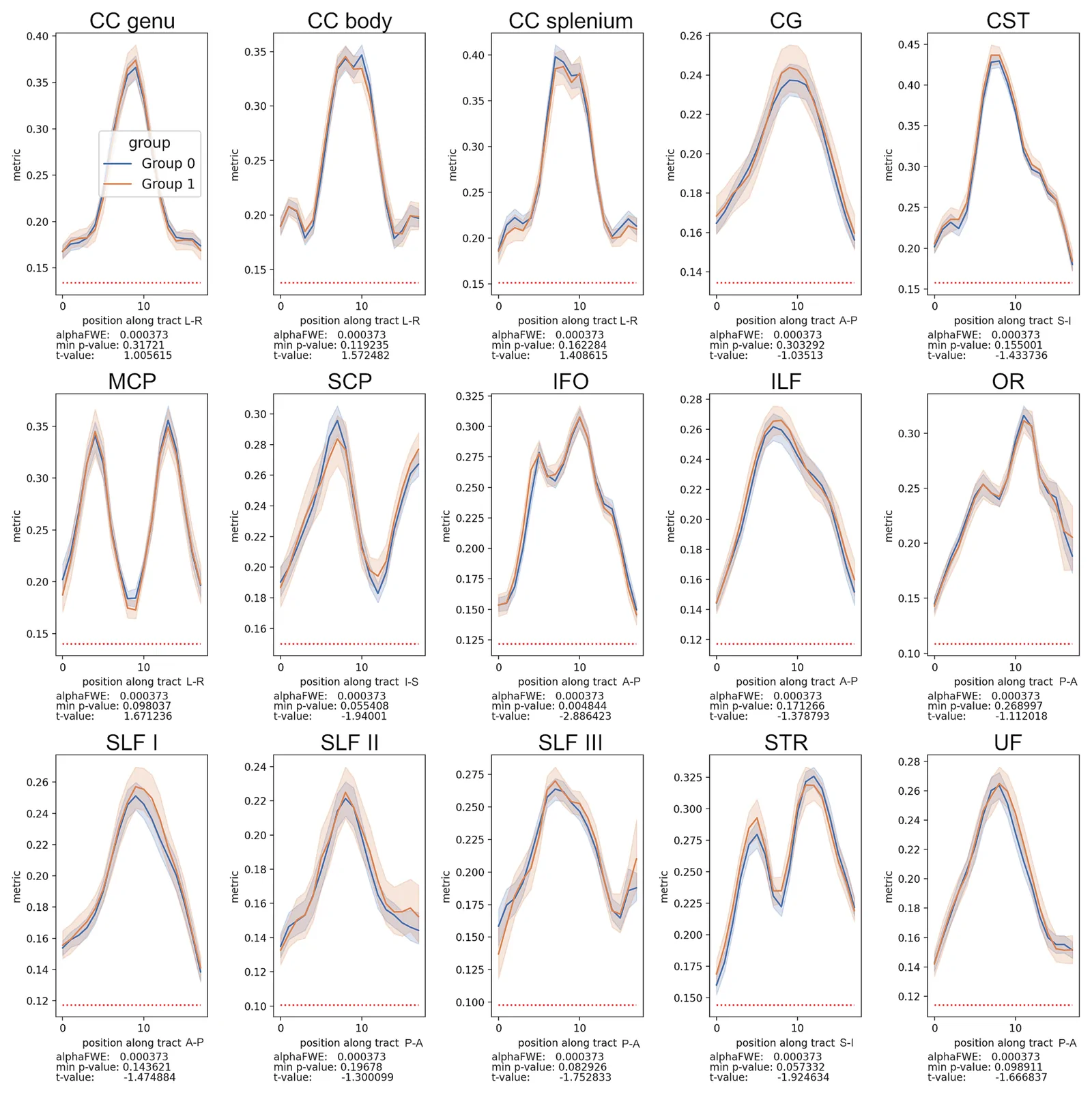
Eight fiber bundle reconstructions using TractSeg in a late preterm infant (35^+^3 weeks’ gestation) scanned at term equivalent age (43^+^3 weeks postmenstrual age) overlaid on the b0 diffusion MRI scan. Directional color coding was applied: red is left–right, blue is inferior-superior, and green is anterior-posterior. (For interpretation of the references to color in this figure legend, the reader is referred to the web version of this article.)

The visual assessment of the reconstructed fiber bundles did not reveal any overt deviations from known neuroanatomy. The anatomical shape, position, and symmetry of the tracts were consistent with expected neuroanatomical structures, with streamlines displaying appropriate continuity and termination points (Fig. 3). Bilateral tracts demonstrated symmetry as anticipated and reconstruction of crossing fiber bundles was visually confirmed.

**Fig. 4:**
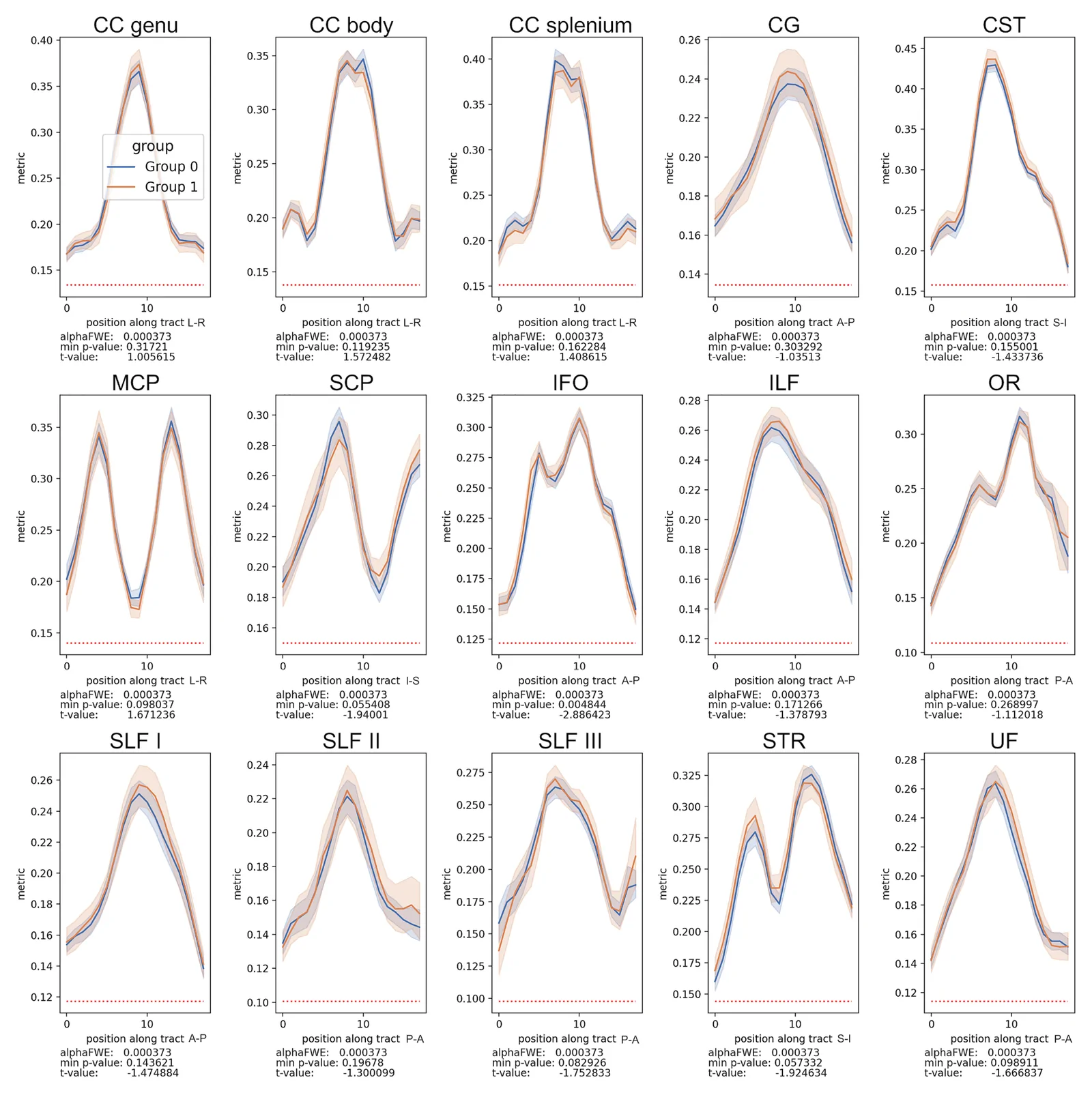
Fractional anisotropy (FA) fiber tract profiles across 15 bundles visualized for late preterm (group 0; blue) and moderate preterm (group 1; orange) infants with correction for multiple testing. The colored shaded areas around the mean provide the 95% confidence interval and the red dotted lines indicate that there are no significant differences between groups. AlphaFWE: significance level, min p-value: minimally found p-value in each bundle, t-value: the difference in group means measured in units of standard error. CC: corpus callosum; CG: cingulum; CST: corticospinal tracts; MCP: middle cerebellar peduncle; SCP: superior cerebellar peduncle; IFO: inferior fronto-occipital fascicle; ILF: inferior longitudinal fascicle; OR: optic radiation; SLF: superior longitudinal fascicle; STR: superior thalamic radiation; UF: uncinated fascicle. The position of the graphs along the direction of the tracts is indicated with A: anterior, P: posterior, L: left, R: right, I: inferior, and S: superior. (For interpretation of the references to color in this figure legend, the reader is referred to the web version of this article.)

There were no statistically significant differences in along-tract diffusion measures between MP and LP infants (Fig. 4). However, along-tract profiles exhibited alternating patterns of FA and MD differences between the groups, accompanied by wide confidence intervals for both groups. For most bundles, FA profiles alternated between MP or LP infants, with each group showing higher FA values in different segments. In the superior cerebellar peduncle and superior thalamic radiation, a shift occurred over the course of the tract, where the group with higher FA values reversed. MP infants showed higher FA values along the majority of the cingulum bundle and the inferior and superior longitudinal fascicles. In contrast, LP infants only showed higher FA values along the majority of the splenium of the corpus callosum. The corticospinal tracts, included as control bundle, showed consistent FA profiles between MP and LP infants, with narrow confidence intervals (Fig. 4).

Similar to the FA tract profiles, MD profiles demonstrated alternating differences between MP and LP infants. The cingulum and superior longitudinal fascicle I showed non-significant yet distinct differences along their entire length, also with wide confidence intervals. Tract profile plots and statistical results for FA and MD with and without correction for multiple comparisons are provided in the supplemental material (Figs. S1-S4).

## 4 Discussion

This study is the first to analyze tractography-based diffusion measures in relatively healthy MP and LP infants without visible brain lesions using TractSeg and Tractometry. Along-tract profiles of 15 fiber bundles were generated and compared between MP and LP infants. This study showed feasibility of this deep learning algorithm, trained on adult data, for fiber bundle reconstruction in a neonatal population, including reconstruction of crossing fiber bundles and along-tract measurements. While alternating differences in FA and MD tract profiles between MP and LP infants were detected, suggesting maturational differences between groups, no statistically significant differences were found.

The along-tract profiles reconstructed in this study closely followed the trajectories reported for adults [31] and children aged 8 to 12 years [32]. In MLPT infants, the FA profiles appeared smoother across the bundle length compared to those observed at older ages. This increased smoothness may partially result from dividing each bundle into 20 segments instead of 100. The smaller number of segments was chosen to better suit the smaller neonatal brain’s size and therewith length of tracts, and to balance resolution, smoothness, computational efficiency and number of multiple comparisons. The choice of 20 segments may have introduced a slight averaging effect, as each segment spans approximately 2 or 3 voxels along the tract. As expected, the FA values in MLPT infants were lower compared to FA values at an older age, reflecting the reduced level of maturation at this developmental stage. Furthermore, the narrow confidence intervals observed in the corticospinal tract profiles, used as a control, suggest consistency and reliability of the methods employed for along-tract profiling of diffusion measures. These findings suggest that deep learning techniques, such as TractSeg, can also be used for tractography in this neonatal population. However, it is important to note that TractSeg was originally trained on adult data, and the structural and microstructural differences between adults and infants may have influenced the reliability of tract segmentation [26]. Despite this, TractSeg has been tested on a range of held-out datasets (i.e., data that was not used for training the tool), both with and without pathologies, demonstrating its adaptability to pathological differences in the adult brain [26]. This evidence supports the notion that TractSeg is likely adaptable to the smaller, less developed neonatal brain as well.

Although diffusion measures were not statistically significantly different, alternating differences in tract profiles were observed between MP and LP infants. The corticospinal tracts, serving as control, showed good similarity between the groups with small confidence intervals. In contrast, most other tracts showed greater differences and larger confidence intervals, suggesting developmental differences in brain microstructure between and within MP and LP groups. Interestingly, bundles with higher FA values, suggesting advanced maturity, in MP infants were more often related to cognitive, language and visual functions, while bundles with higher FA values in LP infants were more often related to motor function. The higher FA values observed in MP infants may be related to their longer exposure to the extra-uterine environment and increased stimuli of visual, linguistic and cognitive pathways. This may suggest that ex-utero exposures may play a role in the development of these neural pathways. However, results were not statistically significant, and caution with interpretation is warranted. In addition, an increased FA value does not inherently indicate better microstructural development in MP infants [33]. As the brain matures and establishes more complex connections, such as an increase in streamline crossings, intra-voxel orientational coherence decreases, potentially resulting in a reduction in FA [33]. Therefore, observed differences in diffusion measures, including lower FA, may reflect normal developmental processes rather than indicating delayed development [33]. As shown in a review by Pannek et al., covering a broad GA range, the relation between GA at birth and diffusion metrics remains unclear [34]. A more recent study on sensorimotor tracts did find statistically significant FA and MD differences between very preterm and full term infants, but not between MLPT and full term infants [35]. Together, these findings highlight the need for continued research into the impact of GA as such and related early extra-uterine exposures on neurodevelopment.

The wide confidence intervals for FA and MD across both groups indicate large uncertainty and variability in diffusion measures within groups. This potentially suggests a variability in neurodevelopment within groups, and for other perinatal factors (such as pregnancy and birth complications, environmental factors and genetics) to additionally impact early microstructural development rather than GA. While examining the relationships between these factors and TEA diffusion measures was beyond the scope of our study, such analyses could offer valuable insights into brain developmental differences in the MLPT population.

The few prior studies that investigated microstructural brain development in MLPT infants using dMRI, all applied tract based spatial statistics (TBSS) for analyses [36]. This tool consecutively registers individual brain scans, generates an average FA map, and creates an average FA-skeleton from that map, including all major white matter tracts [37]. The FA-skeleton is then used for voxel-wise comparison. Thompson et al. [38] compared FA, MD, radial and axial diffusivity between MP and LP infants, but found little evidence for microstructural differences between groups [38]. However, when comparing MP and LP infants with full term infants, significantly lower FA and higher MD values were found throughout the white matter skeleton. Additionally, higher FA with lower MD values were found in cerebellar and brainstem regions, cerebral peduncle and internal capsule in the MLPT infants [38]. Kelly et al. [39], in the same cohort, also reported lower FA values in ~ 59 % of white matter skeleton voxels in MLPT infants as compared to full term infants, but no higher FA values [39]. Direct comparisons between our findings and the studies employing TBSS are difficult due to differences in both methodological and analytical approaches, and study populations (e.g. inclusion of infants with brain lesions and comparison to full term infants). Specifically, TBSS studies involve voxel-based comparisons and often rely on FA skeleton thresholding, which may suppress subtle differences. Opposed to common dMRI studies in MLPT infants, the inclusion of higher b-values in our cohort enabled the analysis of more complex tract configurations and was therefore chosen over TBSS analysis.

As we recently highlighted, few prior studies have employed tractography to investigate developmental differences in white matter microstructure between infants with and without brain lesions and between neonatal populations, such as preterm (25–36 weeks’ gestation) versus full-term infants [12]. Although these studies did not distinguish preterm subgroups, they have highlighted less advanced development of the preterm brain as compared to the full-term brain [12]. Tractography has also previously been used to assess spatiotemporal developmental patterns from birth up to TEA in preterm and full-term infants, describing lateral-to-medial thalamic and posterior-to anterior thalamocortical developmental patterns [40]. In addition, similar tools have been applied in preterm cohorts to investigate the brain connectome in comparison to full-term infants, showing a topological reorganization of the structural network during the perinatal period, where local topology was more vulnerable to premature birth than global topology [41,42]. While direct comparison of studies is challenged by methodological differences, such as population, imaging settings and analysis approach, these studies, along with the studies investigating comparable cohorts that apply TBSS analyses, strengthen our hypothesis and findings of developmental differences in brain white matter microstructure related to prematurity. In addition, they highlight the importance of further studies to advance our understanding of preterm brain development, crucial to improving clinical care for these vulnerable infants.

### 4.1 Strengths and limitations

We were the first to apply TractSeg with Tractometry for tractography-based along-tract profiling in MP and LP infants, and demonstrated its feasibility for visualizing complex, crossing tracts in the neonatal brain with high b-value dMRI data. Some limitations should be addressed. We included a relatively small sample size, which may have contributed to the large confidence intervals. Future studies with larger cohorts are recommended to identify potential subtle differences between groups. In addition, we did not include a control group of full term infants. A comparison of diffusion measurements of MLPT infants with those in full term infants at TEA could have revealed differences in brain development and diffusion measures. Finally, in preterm research, it is common to correct for PMA at scan, as variation in brain development can occur due to being an older age at scan time [43]. However, testing on our cohort revealed that PMA was not a confounding factor and was therefore not included in the analyses.

### 4.2 Future directions

Despite the growing interest in understanding brain development in relation to neurodevelopmental challenges in MLPT infants, research on diffusion measures in this population remains limited, hampering early identification of at-risk MLPT infants and the development of neuroprotective strategies. To gain a more comprehensive understanding of white matter developmental differences between MP and LP infants, and the MLPT population overall, further research is essential. Future studies should explore advanced diffusion MRI techniques, such as more microstructure-specific models beyond the diffusion tensor, additional tract-based comparisons or connectome-based analyses, to investigate whole brain structural connectivity and network organization. In addition, efforts should be made to establish a consensus on dMRI data analysis methodologies. A unified approach would enable more robust comparisons across studies, facilitating a deeper understanding of developmental trajectories.

Though not statistically significant, the variability in diffusion measures between MP and LP infants in this study shows promise to better understanding the underlying mechanisms of later neuro-developmental challenges in MLPT infants. The absence of statistically significant findings may be explained by small age and therewith maturation and risk profile differences between being born MP (up to 33^+^6 weeks’ gestation) versus LP (from 34^+^0 weeks’ gestation) and reaching TEA. Developmental risks are believed to extend continuously across the GA spectrum, suggesting that these cutoffs may not fully capture the nuances of risk associated with each week or day of gestation [44]. Exploring correlations between along-tract diffusion measures and GA as a continuous variable, and mapping diffusion measures in healthy full term infants may further enhance our understanding of early brain development. Additionally, alterations in brain development induced by being born a few days or weeks earlier might not have caused noticeable differences at this early stage. Longitudinal studies tracking brain development from infancy through early childhood and beyond may bring to light how early diffusional differences evolve and potentially relate to later neurodevelopmental outcomes. Moreover, clinical parameters – such as perinatal complications, nutrition and medical interventions – may have a greater impact on brain development at this stage than GA alone. Investigating how these clinical and environmental parameters influence diffusion measures at TEA may reveal modifiable risk factors to optimize brain development. Moving beyond this, developing a predictive model incorporating clinical, environmental and imaging parameters may further enhance our ability to identify infants at increased risk for developmental problems.

## 6 Conclusion

Deep learning-based tractography was shown to be feasible on dMRI data from MLPT infants, allowing reproducible reconstruction of crossing fiber bundles. Along-tract profiles were consistent, reliable, and in line with expectations based on tract profiles from adult subjects. Though not statistically significant, the alternating differences in FA and MD tract profiles between the two groups suggest that subtle developmental variability may exist. Given the complexity and under-researched nature of brain development in MLPT infants, these findings highlight the need for further investigation. Future studies are essential to better understand white matter maturation in this population and to elucidate the potential long-term implications of early developmental variability.

## Supplementary Material

Supplementary data to this article can be found online at https://doi.org/10.1016/j.ejrad.2025.112098.

Supplementary data

## Figures and Tables

**Table 1:** Participant characteristics.

	MP	LP	p-value	Excluded	p-value^a^
	n = 31	n = 64		n = 43	
**Neonatal period**					
Gestational age in weeks, median (range)	33.1 (32.0–33.9)	35.3 (34.0–35.9)	**<** **0.001**	34.6 (31.6–35.9)	0.77
Birth weight in grams, mean (SD)	1973 (296)	2361 (382)	**<** **0.001**	2217 (365)	0.81
Head circumference in cm, mean (SD)	30.6 (1.5)	32.2 (1.5)	**<** **0.001**	31.5 (1.7)	0.49
Sex, n (%)			0.99		>0.99
Male	14 (45)	29 (45)		19 (44)	
Female	17 (55)	35 (55)		23 (53)	
Plurality, n (%)			0.41		0.34
Singleton	26 (84)	49 (77)		30 (70)	
Twin	5 (16)	15 (23)		12 (28)	
Admission level, n (%)			0.78		0.87
No admission	23 (74)	51 (80)		31 (72)	
Level 2	7 (23)	12 (19)		10 (23)	
Level 3	1 (3)	1 (1.5)		1 (2)	
Small for gestational age (third percentile), n (%)	1 (3)	3 (5)	0.739	–	–
Continuous positive airway pressure, n (%)	22 (71)	26 (41)	**0.006**	–	–
Suspicion of early onset sepsis, n (%)	21 (68)	20 (31)	**0.003**	–	–
Meningitis, n (%)	0 (0)	0 (0)	–	–	–
Bronchopulmonary dysplasia, n (%)	2 (6)	0 (0)	**0.04**	–	–
Necrotizing Enterocolitis, n (%)	0 (0)	0 (0)	–	–	–
Hemodynamically significant patent ductus arteriosus, n (%)	0 (0)	0 (0)	–	–	–
Hypoxic-ischemic encephalopathy, n (%)	0 (0)	0 (0)	–	–	–
**Characteristics at TEA MRI**					
PMA at MRI in weeks, median (range)	41.3 (40.3–45.7)	41.8 (38.7–44.7)	0.50	41.8 (39.1–44.7)^e^	0.60
Weight in grams, mean (SD)	3404 (554)^b^	3577 (590)	0.18	3406 (553)^e^	0.36
Head circumference in cm, mean (SD)	35.7 (1.3)^c^	35.3 (1.2)^d^	0.20	35.6 (1.1)^f^	0.51

n: number; SD: standard deviation; TEA: term equivalent age; PMA: postmenstrual age

a p-values for included vs. excluded

b n = 29

c n = 26

d n = 52

e n = 26

f n = 25.

Bolded values are significant.
