# Supplementary material for: Diffusion MRI tractography with along-tract profiling reveals subtle neurodevelopmental differences between moderate and late preterm infants
Source: Eur J Radiol. Author manuscript; Available in PMC 2026 Nov 11. (PMC7619721; doi:10.1016/j.ejrad.2025.112098)
Supplement: Supplementary file 1 [file EMS221410-supplement-Supplementary_data.docx]

*Figure S1: Fractional anisotropy (FA) fiber tract profiles of 15 bundles visualized for late preterm (group 0; blue) and moderate preterm (group 1; orange) infants without correction for multiple testing. The position of the graphs along the direction of the tracts is indicated with A: anterior, P: posterior, L: left, R: right, I: inferior, and S: superior.*

*Figure S2: Fractional anisotropy (FA) fiber tract profiles of 15 bundles visualized for late preterm (group 0; blue) and moderate preterm (group 1; orange) infants with correction for multiple testing. The position of the graphs along the direction of the tracts is indicated with A: anterior, P: posterior, L: left, R: right, I: inferior, and S: superior.*

*Figure S3: Mean Diffusivity (MD) fiber tract profiles of 15 bundles visualized for late preterm (group 0; blue) and moderate preterm (group 1; orange) infants without correction for multiple testing. The position of the graphs along the direction of the tracts is indicated with A: anterior, P: posterior, L: left, R: right, I: inferior, and S: superior.*

*Figure S4: Mean Diffusivity (MD) fiber tract profiles of 15 bundles visualized for late preterm (group 0; blue) and moderate preterm (group 1; orange) infants with correction for multiple testing. The position of the graphs along the direction of the tracts is indicated with A: anterior, P: posterior, L: left, R: right, I: inferior, and S: superior.*
